# Organizational culture and turnover intention among primary care providers: a multilevel study in four large cities in China

**DOI:** 10.1080/16549716.2024.2346203

**Published:** 2024-06-03

**Authors:** Mengyao Li, Wenhua Wang, Jinnan Zhang, Ruixue Zhao, Katya Loban, Huiyun Yang, Rebecca Mitchell

**Affiliations:** a School of Public Policy and Administration, Xi’an Jiaotong University, Xi’an, People’s Republic of China; b Research Institute of the McGill University Health Centre, McGill University, Montreal, Canada; c Health and Wellbeing Research Unit (HoWRU), Macquarie Business School, Macquarie University, Sydney, Australia; d Newcastle Business School, University of Newcastle, Newcastle, Australia

**Keywords:** Turnover intention, organizational culture, primary health care, primary care providers, organizational management

## Abstract

**Background:**

Primary health care plays an important role in providing populations with access to health care. However, it is currently facing unprecedented workforce shortages and high turnover worldwide.

**Objective:**

This study examined the relationship between organizational culture and turnover intention among primary care providers in China.

**Methods:**

A cross-sectional survey was administered in four large cities in China, Tianjin, Jinan, Shanghai, and Shenzhen, comprising 38 community health centers and 399 primary care providers. Organizational culture was measured using the Competing Value Framework model, which is divided into four culture types: group, development, hierarchy, and rational culture. Turnover intention was measured using one item assessing participants’ intention to leave their current position in the following year. We compared the turnover intention among different organizational culture types using a Chi-square test, while the hierarchical logistic regression was used to examine the relationship between organizational culture and turnover intention.

**Results:**

The study found that 32% of primary care providers indicated an intention to leave. Primary care providers working in a hierarchical culture reported higher turnover intention (43.18%) compared with those in other cultures (*p* < 0.05). Hierarchical culture was a predictor of turnover intention (OR = 3.453, *p* < 0.001), whereas rational culture had a negative effect on turnover intention (OR = 0.319, *p* < 0.05).

**Conclusions:**

Our findings inform organizational management strategies to retain a healthy workforce in primary health care.

## Paper Context

- **Main findings:** This study found that primary care physicians and nurses working in a hierarchical culture are more likely to report the intention to leave compared to other culture types, while those working in a rational culture are significantly less likely to report the intention to leave.
- **Added knowledge:** The dominant organizational culture identified in community health centers across eastern China is group culture, and organizational culture is a significant predictor of the turnover intention of primary care providers.
- **Global health impact for policy and action:** Future primary care reform should focus on managerial interventions in their efforts to retain health workers and, in particular, develop and implement strategies to cultivate and moderate rational culture.

## Background

Health workforce shortages are a global challenge, and it is estimated that a worldwide shortage of health workers will result in 15 million unfilled vacancies by 2030 [1]. To address this challenge, the World Health Organization (WHO) has put forward a series of recommendations to train, attract, recruit, and retain health workforce, with a specific focus on primary care [2]. Since 2009, China has implemented a series of health reforms, with considerable support for primary health care (PHC), including measures to train, attract and retain primary care providers (PCPs) to provide its population with high-quality health care [3]. For example, in 2015, a new ‘5 + 3’ training program was developed to improve the training of qualified general practitioners involving five years of medical school education and three years of standardized residency training [4]. In 2010, a tuition fee waiver program was implemented to support medical students who agreed to serve at least six years in a local PHC institution after graduation [5]. Additional support was also provided to train nurse practitioners to fulfill some of the roles traditionally held by physicians, although this initiative is still in the exploratory stage [6]. Recently, the national blueprint ‘The Healthy China 2030 Plan’ re-emphasized the value of PHC, and the importance of PCPs [7]. The achievements of the PHC health workforce reforms are notable as, from 2009 to 2020, the number of health professionals in PHC rose from 1.83 million to 3.12 million [8,9]. The Chinese government has set a national target of five general practitioners per 10 000 population by 2030 [10]; however, currently there are 2.9 general practitioners per 10 000 population [9]. New strategies are needed to train more primary care professionals and to retain them in primary care.

The high turnover intention among PCPs is a global challenge [11]. Previous studies have suggested that 25% of PCPs in the USA [12], 41.9% of the general practitioners in England [13], and 51.1% of nurses in primary care clinics in South Africa [14] expressed an intention to leave their current job. A recent systematic review showed that the turnover intention of primary care workers in China was 30.4% [15]. Turnover intention is a strong predictor of actual turnover [16,17], and high level of turnover intention not only leads to potential primary care workforce shortage but also influences health care performance and cost. For example, the turnover intention of nurses was found to be negatively related to self-reported quality of care [18], including failure to respond to patient requests in a timely manner and poor understanding of patients’ thoughts and feelings. The turnover intention of PCPs can also lead to significant financial costs, and research conducted in 2017–2018 in the USA indicated that turnover of PCPs resulted in approximately $979 million extra health care expenditures [12]. Thus, reducing turnover intention among PCPs is a crucial step towards maintaining a robust workforce and reducing health care costs in primary care.

In China, the PHC system delivers general clinical care and basic public health services. Over 90% of primary care facilities are publicly owned. Community Health Centers (CHCs) and Community Health Stations serve as the principal facilities providing primary care services in urban areas, with CHCs assuming a leading role [11]. In 2019, the National Health Commission of China first issued guidelines for evaluating the service capacity of CHCs, covering functional mandates and resourcing, basic medical and public health services, operational management, and comprehensive management [19]. These goals and indicators, established at the national level, have been adapted and are being implemented at the provincial level and district level.

Numerous studies have explored the factors affecting turnover intention in PHC. A recent systematic review categorized antecedents of turnover intention into demographic characteristics (gender, age, education, marital status), job characteristics (remuneration, social status, organizational affiliation, work stress), and job satisfaction characteristics (development opportunity, interpersonal relationships, etc.) [15]. Almost all of this research has focused on PCP factors, job factors, and the interaction between health care providers and work environment in PHC [13,14,20–25]. These studies have focused on the individual PCPs and have resulted in individual-level recommendations to reduce turnover intention. Conversely, the body of literature on organizational-level influences on turnover intention is more limited. While research has analyzed the influence of organizational factors, such as leadership, organizational structure and financial incentives on turnover intention among nurses [26,27], the role of other ‘soft’ organizational factors has received less attention. In particular, the role of PHC organizational culture remains unclear. This is a surprising omission as previous research suggests that CHC culture is well developed and influential with PCPs reported as working within an atmosphere of shared vision, values and beliefs reflective of organizational culture that influences job satisfaction, strongly predicting turnover intention [28]. The organizational culture within CHCs in China is characterized by rigidity and collaborative teamwork, which fosters a harmonious atmosphere and team cohesion. However, this cohesion may be perceived as a form of militarized cohesion within a hierarchical structure [29]. Further, there is consistent evidence that organizational culture affects turnover intention across various industries and sectors, including the government sector [30] and child welfare institutions [31]. In health care, it has been shown that a common determinant of turnover intention among physicians is the mismatch between their expectations and the organizational culture [32]. However, these studies were conducted in hospital settings [33,34], and the influence of culture on turnover intention in CHCs remains poorly understood.

The aim of this study was to understand the type of organizational culture that exists in CHCs in China and to study the relationship between organizational culture and turnover intentions of PCPs within CHCs. Given existing findings related to organizational culture antecedents [35], the results of this study may provide useful information for frontline primary care managers regarding specific management levers to retain PCPs in China and beyond, as well as informing further primary care workforce reform and policy making.

### Conceptual framework

This study examined the relationship between the organizational culture of CHCs and the turnover intention of PCPs, using the Competing values framework (CVF) which has been frequently applied to assess organizational culture in health services research [30,31,36]. It is a useful model for understanding the organizational factors, including leadership roles and financial strategy [36–38]. Previous research has linked management styles and financial strategies to nurses’ decisions to exit their jobs, noting that leadership influenced turnover mediated by job satisfaction [26,27].

The CVF model has two dimensions of organizational structure and organizational focus, the former emphasizing control and stability of organizational processes as well as flexibility and dynamism, and the latter referring to internal orientation, solidarity, external orientation and competition [36,39]. Spanning these two dimensions are four types of culture: group culture (emphasis on teamwork and development of human resources), development culture (emphasis on expansion, creativity and innovation), hierarchical culture (emphasis on stability and adhering to rules), and rational culture (emphasis on tasks, goals, and outcomes).

Turnover intention is an individual’s willingness or plan to quit their current job [40], which involves several steps, including intent to resign, search for a new job, and securing a new job [40,41]. Early turnover research focused on the central constructs of movement desirability and ease [42]. From this research, job satisfaction and job opportunity became cornerstones of contemporary turnover models [41,43]. As the status of turnover intention is considered to be a strong predictor of turnover and its measurability [20], this research analyzed turnover intention as the key outcome variable. We assessed personal demographics, organizational characteristics and work condition factors related to turnover intention, and included burnout, work-family conflict, and organizational commitment as covariates [15,44–46].

## Methods

### Study setting

This study was conducted in 38 CHCs in four large cities in China: Tianjin, Jinan, Shanghai, and Shenzhen. These four cities are in the east of China, with evidence of the highest prevalence of turnover intention [15]. In accordance with the sample size requirements for statistical analysis [47] and considering the constraints of the COVID-19 situation, we sampled 38 CHCs including 399 PCPs. The selection of 38 CHCs across four cities was guided by recommendations from local health bureau staff, who considered a range of performance levels and ownership types to ensure a diverse representation of CHCs.

### Participants and data collection

A cross-sectional survey using the convenience sampling approach was administered in four cities to 38 directors of CHCs and 399 PCPs (224 physicians and 175 registered nurses). The directors completed the organization survey on CHC characteristics, including the organizational size, organizational ownership, and organizational culture, given the directors’ role in providing organizational oversight and managing human resources [33]. The PCPs completed a professional survey including turnover intention, burnout, organizational commitment, work-family conflict, and personal characteristics.

The study was carried out from November 2021 to May 2022. Paper questionnaires were handed out to participants face-to-face with the goal of obtaining high-quality data. The researchers clarified the questionnaire wording to the participants, instructing them to complete it honestly and in accordance with their true feelings. All subjects provided informed consent then filled out the questionnaires anonymously. All 399 questionnaires were collected, one of which was subsequently excluded due to missing information. Study ethics approval was obtained from the Ethics Committee of Xi’an Jiaotong University.

### Measures and variables

#### Outcome variable

This study used one item in the questionnaire to measure turnover intention: ‘I have intention to leave my current workplace in the next year’, to which participants responded on a 5-point Likert scale, with 1 representing strongly disagree and 5 representing strongly agree. The 5-point Likert scale was subsequently collapsed into a binary category, namely disagree (strongly disagree, disagree) represented by 0 and agree (average, agree, strongly agree) represented by 1 [48]. The mean value of the turnover intention variable indicated the percentage of PCPs who intended to leave their current workplace, with higher values indicating higher turnover intention.

#### Explanatory variable

Organizational culture was the key explanatory variable. To measure culture, we used the 20-item version of the CVF instrument, with established reliability [49]. To ensure the feasibility and reliability in the Chinese context, we translated and refined the instrument through consultations with senior primary care researchers, local primary care directors and physicians. The instrument has five groups of statements (four statements per group) representing the following five aspects of the organization: major characteristics, leadership style, organizational cohesion, organizational focus, and organizational rewards [50]. Participants were asked to allocate 100 points to the four statements in each group, with ‘A’ for group culture, ‘B’ for development culture, ‘C’ for hierarchical culture, and ‘D’ for rational culture. For each type of culture, the average score of the statements was calculated. The culture type with the highest score was assessed as the dominant culture type in the CHC – the explanatory categorical variable used in subsequent analyses. Cronbach’s alpha coefficients were 0.61 for group culture, 0.42 for development culture, 0.82 for hierarchical culture, and 0.35 for rational culture. These relatively low values could be attributed to the limited sample size of 38 CHCs. However, the Cronbach’s alpha values are consistent with other organizational culture studies, where the minimum value was 0.40 [49,51].

#### Covariates

Covariates in this study included three groups: personal demographics, organizational characteristics, and work condition factors associated with turnover intention. Personal demographics were: age, sex (female, male), marital status (married, unmarried), educational background (high school or below, junior college and above), and years of work experience. Organizational characteristics included organizational size (number of staff) and organizational ownership (government-managed CHCs or public hospital-managed CHCs). Work condition factors associated with turnover intention were informed by previous research [44–46] and included three variables: burnout, work-family conflict, and organizational commitment.

We measured burnout – the emotional exhaustion of PCPs, with four items using a 5-point Likert scale (1 = strongly disagree, 5 = strongly agree), and the internal consistency test showed a Cronbach alpha of 0.86 in the previous research [52]. Work-family conflict was measured with four items using a 7-point Likert scale (1 = strongly disagree, 7 = strongly agree), which had high reliability with a Cronbach's alpha of 0.83 in the previous research [53]. Organizational commitment was measured with six items using a 5-point Likert scale (1 = strongly disagree, 5 = strongly agree) and showed high reliability with a Cronbach's alpha of 0.82 in prior research [54]. Average scores for the three variables were subsequently calculated with higher averages signifying greater variable strength. The scales measuring burnout, work-family conflict, and organizational commitment in this survey demonstrated high reliability, as evidenced by a Cronbach's alpha coefficient of 0.87, 0.91, and 0.95, respectively, in the current research.

### Statistical analyses

First, descriptive statistical analyses were performed, including the frequency (N) and percentage (%) of socio-demographic, work-related, and CHC-related characteristics. Second, we calculated the mean (M) and standard deviation (SD) and assessed correlation and reliability of the study variables. Pearson correlation coefficient and Spearman correlation coefficient for categorical variables were used to test the correlation of the study variables. Next, Chi-square tests were conducted to compare different CHC culture types on participant intention to leave their current job. Finally, hierarchical logistic regression modelling was undertaken to examine the relationship between the organizational culture and turnover intention, while taking into account the impact of the covariates. In models 1 through 4, a single organizational culture type was identified as the independent variable, with the other remaining culture types serving as references. In model 1, the independent variable was group culture, and the reference was non-group culture. The objective was to investigate the disparities in turnover intention among physicians and nurses operating within group culture as compared to those in non-group culture.

We used hierarchical logistic regression modelling because it is an appropriate model for nested data, that is, PCPs nested in their respective CHCs in this study [55]. All variables in our study, excluding categorical variables, were centered at the grand mean to avoid multicollinearity and to facilitate interpretation of the results [56]. We used intraclass correlation coefficient (ICC) as an indicator reflecting the percentage of variation in physicians and nurses intending to leave attributed to CHC-level factors. ICC higher than 0.059 indicates that the hierarchical logistic regression was appropriate to assess the contribution of CHC-level factors to the outcome variable [57]. In addition, we used the variance inflation factor (VIF) and tolerance values to measure collinearity. All statistical analyses were performed using Stata 15.1.

## Results

Table 1 describes the basic characteristics of PCPs and affiliated CHCs. Our final study sample included 398 participants from 38 CHCs: 224 physicians and 174 nurses. Of those, 76.63% were female, and the percentage of female nurses was 98.28%. Only 3.27% of the participants were older than 50. Over 80% of the participants were married or cohabiting. Almost 95% of the participants had completed high school education. Years of working were relatively balanced, with 34.32% working less than 5 years and 38.84% more than 10 years. More than half of the participants worked 41–49 hours per week. The common organizational size of the CHCs in our study was less than 35 staff. Among the 38 CHCs, 60.53% were managed by the government, and the other ones were hospital-managed.

**Table 1.** Descriptive statistical data of the participants and affiliated CHCs.

	Overall398		Physicians224		Nurses174	
	n	%	n	%	n	%
**Individual characteristics (*N* = 398)**						
Sex						
Female	305	76.63	134	59.82	171	98.28
Male	93	23.37	90	40.18	3	1.72
Age						
<30	130	32.66	62	27.68	68	39.08
31–40	168	42.21	99	44.20	69	39.66
41–50	87	21.86	54	24.11	33	18.97
>50	13	3.27	9	4.02	4	2.30
Marital status						
Unmarried	75	18.84	50	22.32	25	14.37
Married	323	81.16	174	77.68	149	85.63
Education						
High school or below	21	5.28	9	4.02	12	6.90
Junior college and above	377	94.72	215	95.98	162	93.10
Years of working						
<5	137	34.42	83	37.05	54	31.03
6–10	108	27.14	53	23.66	55	31.61
>10	153	38.44	88	39.29	65	37.36
Working hours per week						
<40	93	23.37	44	19.64	49	28.16
41–49	218	54.77	123	54.91	95	54.60
50–56	39	9.80	27	12.05	12	6.90
>57	48	12.06	30	13.39	18	10.34
	n			%		
**Organizational characteristics (*N* = 38)**						
Organizational size						
≤35	13			34.21		
36–55	7			18.42		
56–100	7			18.42		
>100	11			28.95		
Ownership						
Government	23			60.53		
Public hospital	15			39.47		

### Correlations among the study variables

Table 2 displays the means and standard deviations, correlations, and the reliability of the study variables. The average scores for burnout, work-family conflict, and organizational commitment were 2.47, 4.02, and 3.98, respectively. The mean of turnover intention was 0.32, indicating that 32% of physicians and nurses intended to leave their current job. Burnout and work-family conflict were negatively correlated with turnover intention (*p* < 0.001), while organizational commitment was positively associated with turnover intention (*p* < 0.001). Organizational commitment was negatively correlated with burnout and work-family conflict (*p* < 0.001), whereas burnout was positively associated with work-family conflict (*p* < 0.001).

**Table 2.** Mean (*M*), standard deviations (*SD*), correlations, and reliabilities of the variables.

		*M ± SD*	1	2	3	4	5	6	7	8	9	10	11	12
1	Turnover intention^a^	0.32 ± 0.47	—											
2	Sex	35.02 ± 7.81	−0.097	—										
3	Gender^a^	0.23 ± 0.42	−0.024	0.058	—									
4	Marital status^a^	0.81 ± 0.39	−0.067	0.398***	−0.022	—								
5	Education^a^	0.95 ± 0.22	−0.030	−0.039	0.024	−0.056	—							
6	Years of working	10.90 ± 8.46	−0.097	0.837***	0.001	0.403***	−0.092	—						
7	Working hours per week	47.14 ± 9.91	0.064	−0.016	0.113*	−0.009	0.020	−0.073	—					
8	Ownership^a^	1.37 ± 0.48	0.064	0.021	0.082	−0.15**	0.157**	−0.122*	0.151**	—				
9	Organizational size	80.32 ± 67.16	−0.028	0.024	−0.093	0.052	−0.034	0.090*	−0.170***	−0.576***	—			
10	Burnout	2.47 ± 1.01	0.335***	−0.034	0.023	0.051	0.008	0.023	0.099**	−0.074	0.024	0.87		
11	Work-family conflict	4.02 ± 1.63	0.224***	0.120**	0.062	0.105*	−0.041	0.125**	0.242***	0.007	−0.038	0.650***	0.91	
12	Organizational commitment	3.98 ± 0.93	−0.321***	0.194***	0.017	0.119*	−0.018	0.202***	0.009	−0.004	0.018	−0.446***	−0.342***	0.95

Note: *N* = 398. Turnover intention: disagree to leave = 0, agree to leave = 1. Sex: female = 0, male = 1. Marital status: not married = 0, married = 1. Education: higher school or below = 0, junior college and above = 1. Ownership: government-managed CHCs = 1, public hospital-managed CHCs = 2.

*p < 0.05. **p < 0.01. ***p < 0.001. Reliability coefficients, Cronbach alpha, appear along the diagonal.

^a^Spearman correlation.

### Organizational culture and turnover intention

Table 3 details the percentages of different organizational cultures in 38 CHCs and participants intending to leave in each of the four culture types. The most common organizational culture in CHCs was the group culture (55.26%), followed by hierarchical culture (23.68%), rational culture (13.16%), and development culture (7.89%). Out of these four types of organizational culture, participants in a hierarchical culture had the highest turnover intention (43.18%), followed by group culture (29.70%), rational culture (21.05%) and development culture (11.54%). The Chi-square test indicated that turnover intention under the hierarchical culture was significantly different from the group culture (χ^2^ = 6.37, *p = *0.012), development culture (χ^2^ = 9.23, *p = *0.002), and rational culture (χ^2^ = 6.12, *p = *0.013), and that the group culture was different from the development culture (χ^2^ = 5.28, *p = *0.022). This suggests that CHCs with a hierarchical culture had higher turnover intention among physicians and nurses than other organizational cultures, and that physicians and nurses working in a group culture had higher turnover intention than those in a development culture.

**Table 3.** Organizational culture in 38 CHCs and the turnover intention of 398 physicians and nurses across four organizational cultures.

	Overall (*N* = 38)	Not intent to leave	Intent to leave	Percent of the intention to leave		
Organizational culture	N (%)	n	n	%	*χ^2^*	*p-*value
Group culture	21 (55.26)	142	60	29.70	15.13	0.002
Development culture	3 (7.89)	23	3	11.54		
Hierarchical culture	9 (23.68)	75	57	43.18		
Rational culture	5 (13.16)	30	8	21.05		

Note: Turnover intention and the percent of the intention to leave are counted by each organizational culture type.

### Hierarchical logistic regression of the factors related to turnover intention

We conducted the collinearity testing prior to regression analysis, and the VIF and tolerance values all met the established criteria. The ICC was 0.1398 indicating that using hierarchical regression was appropriate. Table 4 shows the relationship between organizational culture and turnover intention. From model 1 to model 4, burnout was found to be a positive predictor of intention to leave (*p* < 0.01), and organizational commitment was a negative predictor (*p* < 0.001), whereas work-family conflict was not significantly related to turnover intention. In terms of organizational culture, hierarchical culture was significantly associated with intention to leave (OR = 3.435, *p* < 0.001), implying that employees in CHCs with a hierarchical culture had greater intention to leave compared to CHCs with a non-hierarchical culture. In contrast, CHCs with a rational culture had a significantly lower turnover intention (OR = 0.319, *p* = 0.049). We also conducted a sensitivity analysis using culture as a categorical variable, with hierarchical culture as the reference. The results indicated that the remaining three cultures were all negatively associated with turnover intention (*p* < 0.01), consistent with the result of the Chi-square test. In model 3, employees in hospital-managed CHCs had higher turnover intention than those in government-managed CHCs (OR = 2.310, *p* = 0.042).

**Table 4.** Results of the hierarchical logistic regression analysis.

	Model 1		Model 2		Model 3		Model 4	
	*OR*	95% *CI*	*OR*	95% *CI*	*OR*	95% *CI*	*OR*	95% *CI*
Sex (ref. = female)	0.801	[0.441,1.453]	0.795	[0.438,1.441]	0.804	[0.445,1.452]	0.788	[0.435,1.428]
Age	0.995	[0.934,1.060]	0.992	[0.931,1.056]	1	[0.939,1.065]	0.993	[0.932,1.058]
Marital status (ref = unmarried)	0.761	[0.383,1.509]	0.778	[0.393,1.542]	0.709	[0.359,1.401]	0.714	[0.359,1.421]
Education (ref = high school or below)	0.575	[0.193,1.713]	0.584	[0.198,1.720]	0.605	[0.207,1.767]	0.574	[0.194,1.703]
Years of working	1.003	[0.946,1.064]	1.003	[0.946,1.064]	1.001	[0.945,1.061]	1.006	[0.948,1.067]
Working hours per week	0.993	[0.965,1.022]	0.992	[0.965,1.021]	0.986	[0.959,1.015]	0.993	[0.965,1.021]
Organizational size	0.997	[0.991,1.003]	1.002	[0.994,1.009]	1	[0.994,1.005]	0.997	[0.991,1.003]
Ownership (ref = government-sponsored)	1.498	[0.618,3.629]	1.593	[0.693,3.660]	2.310*	[1.029,5.185]	1.32	[0.586,2.976]
Burnout	1.809***	[1.285,2.548]	1.818***	[1.294,2.556]	1.763**	[1.257,2.473]	1.803***	[1.282,2.536]
Work-family conflict	1.025	[0.827,1.270]	1.024	[0.827,1.269]	1.056	[0.853,1.307]	1.029	[0.830,1.277]
Organizational commitment	0.568***	[0.417,0.776]	0.562***	[0.412,0.766]	0.557***	[0.410,0.758]	0.551***	[0.404,0.752]
Group culture (ref = non-group culture)	0.789	[0.372,1.674]						
Development culture (ref = non-development culture)			0.158	[0.0214,1.168]				
Hierarchical culture (ref = non-hierarchical culture)					3.435***	[1.662,7.098]		
Rational culture (ref = non-rational culture)							0.319*	[0.102,0.998]

Note: OR = Odds ratio. *t* statistics in parentheses. *p < 0.05. **p < 0.01. ***p < 0.001. In model 1, group culture is the key independent variable, and the non-group culture as reference; In model 2, development culture is the key independent variable, and the non- development culture as reference; In model 3, hierarchical culture is the key independent variable, and the non-hierarchical culture as reference; In model 4, rational culture is the key independent culture, and the non-rational culture as the reference.

## Discussion

Using data from 398 PCPs in China, we found that 32% of PCP respondents reported intention to leave and that the dominant organizational culture in CHCs is group culture. We also found that hierarchical culture had a significantly positive association with turnover intention, while rational culture was negatively related to turnover intention. These findings provide useful insights to inform strategies that reduce turnover intention and strengthen the primary care workforce through future primary care reform.

### Turnover intention

Thirty-two percent of the PCPs in this study indicated their intention to leave – a finding that is consistent with results of a systematic review that indicated that turnover intention among primary care workers in China was 30.4% [15]. Research in other countries has found variable degrees of turnover intention among PCPs. A survey of 1,192 general practitioners in England showed that 41.9% intended to leave their practice [13]. Meanwhile, it was conservatively estimated that 25% PCPs in the USA intended to leave their current position [12]. The turnover intention of PCPs in South Africa was 51.1% [14] and in Saudi Arabia it was reported as approximately 40% [25].

### The relationship between organizational culture and turnover intention

Group culture was the most common organizational culture type among CHCs in this study. This finding echoes the conclusions of research conducted among primary care teams in England that identified group culture as the dominant culture type [39]. In China, however, the evidence regarding organizational culture types is sparse, with only one study reporting that Chinese public hospitals exhibited a hierarchical culture with relatively strong rules and regulations [58]. According to the Mintzberg organizational configurations framework [59], hospitals can be classified as a professional bureaucracy which are relatively formalized organizations employing highly trained professionals in complex but relatively stable environments [60], and this may explain the need for more hierarchical approaches. However, the size of professional bureaucracies is usually moderate to large [60], yet the number of beds in a typical CHC in China is less than 20, whereas the number of beds in a tertiary hospital in China is usually over 500. Research conducted in English hospitals indicated that the dominant culture was the group culture, with the average bed size being 675 [61]. This suggests that organizations of similar sizes may exhibit different cultures across different countries. Our finding indicates the multifaceted influences on culture noting that the organizational culture of healthcare institutions is likely to vary in the context of the social environment, as well as differing governance and financing structures within the health system.

Within the CHCs in our study that exhibited features of a hierarchical culture, a significantly higher turnover intention was reported among both PCPs and registered nurses. This type of culture emphasizes stability and adherence to rules, procedures and regulations [36,38]. Our finding is in alignment with a Korean study that identified a higher turnover intention among hospital nurses working in a hierarchical culture [34]. However, an American study conducted in a nursing home setting provided a more nuanced assessment highlighting the variable effect of culture on nurses of different levels, with lower intention to resign among registered nurses working in a hierarchical culture [33]. While our study did not identify the differential impact of the hierarchical culture on primary care physicians and nurses, organizational culture may indeed be experienced differently by different groups of health care providers [33], highlighting the need for further research in this area.

Another important finding from our study is that PCPs working in a rational culture had a significantly lower turnover intention than in other cultures. According to the CVF model, the rational culture is externally oriented, with a stable organizational structure and clear objectives, and emphasizes goal fulfillment and achievement-based rewards [36,38]. It has been demonstrated in prior research that the relationship between the rational culture and turnover intention can fluctuate. A study of Korean public servants demonstrated a U-shaped association, whereby turnover intention weakened when the rational culture was moderate and intensified when the overemphasis on productivity led to employee burnout [30]. China is likely at the initial stage of the U-shape curve, as it is increasingly focusing on strengthening the performance assessment of primary health care institutions, as per the guidelines for evaluating primary care services issued in 2019 [19,62]. This policy of assessing performance sets a clear goal for institutions and PCPs, corresponding with the orientation of a rational culture. Achievement of these organizational goals will likely promote the organizational commitment of PCPs and diminish burnout or dissatisfaction of PCPs, contributing to lower turnover intention.

### Turnover intention and other control factors

Not surprisingly, burnout was positively correlated with turnover intention, whereas organizational commitment was negatively correlated with turnover intention. The association between work-family conflict and turnover intention was not significant. Another study among 3,563 physicians in China indicated that work-family conflict had much greater indirect than direct effects on turnover intention, and that it was challenging to examine direct effects in small samples [46]. In our final regression model, hospital-managed CHCs had a higher turnover intention than the government-managed ones. One of the possible reasons is that community residents may prefer hospital-managed CHCs due to better health services provided by these entities, which, in turn, contributes to additional workload for CHC health care professionals in these establishments [63]. Further research is therefore needed to study the impact of CHC ownership models on turnover intention.

This study has several limitations. First, the study participants were recruited from provinces in the east of China with more developed economies and better infrastructure than other regions in China. Future research could investigate the relationship between turnover intention and organizational cultures in other less-resourced settings to determine the generalizability of our findings to less well-developed health care settings. In addition, CHCs in our study were publicly owned, and caution is warranted when transferring these findings to private CHCs that provide primary care services. Second, given the cross-sectional nature of this study, it is difficult to establish the cause-and-effect relationship between turnover intention and organizational culture. Further studies are recommended that use longitudinal data to directly assess causality. In should be noted that our study was conducted immediately following the COVID-19 pandemic which had a detrimental impact on the health care workforce worldwide. The collection of longitudinal data is also warranted to examine any further change in turnover intention. Finally, years of working was utilized as a proxy for income and identified as a critical factor influencing turnover intention. It is recommended that future research collect direct income data from health workers or institutions to assess the validity of this relationship. It is important to acknowledge that organizational culture is malleable to some degree; therefore, lowering turnover intention or enhancing organizational effectiveness can be achieved by concurrently considering managerial roles, organizational types, and external environmental influences.

### Practice implication

High turnover intention in the primary care workforce is a global challenge. Addressing it is a priority for universal health coverage. Our study found evidence of a novel relationship between organizational culture and turnover intention in primary health care settings, suggesting that retaining PCPs could be facilitated through promoting a moderate rational culture as part of future primary care reform. Policy-makers can consider institution-level features such as organizational values, leadership, rewards and structure in formulating the strategies to retaining health workforce given their role in shaping organizational culture. We call for the development of such strategies to support the retention of the primary care workforce in the future health care reform.

## Conclusion

This study found that the dominant organizational culture in CHCs in China is group culture. Our data indicate that CHCs with a rational culture had significantly lower turnover intention, whereas hierarchical culture was associated with higher turnover intention compared with the other three culture types. These findings can be used to inform strategies to reduce turnover intention and to strengthen the primary care workforce by promoting a rational organizational culture and by shifting or weakening hierarchical cultures. Future research should focus on assessing the differential impact of the same culture type on different health care provider groups and on identifying concrete pathways and mechanisms that explain how culture influences turnover intention.
